# Effectiveness of interventions to prevent drowning among children under age 20 years: a global scoping review

**DOI:** 10.3389/fpubh.2024.1467478

**Published:** 2024-12-31

**Authors:** Lamisa Ashraf, Nukhba Zia, Joanne Vincenten, J. Morag Mackay, Priyanka Agrawal, Abigail Green, Abdulgafoor M. Bachani

**Affiliations:** ^1^Johns Hopkins International Injury Research Unit, Health Systems Program, Department of International Health, Johns Hopkins Bloomberg School of Public Health, Baltimore, MD, United States; ^2^United Nations International Children’s Emergency Fund, New York, NY, United States; ^3^Safe Kids Worldwide, Washington, DC, United States

**Keywords:** drowning, children, adolescents, prevention, interventions, evidence synthesis, effectiveness levels, sustainable development goals

## Abstract

**Background:**

Drowning is a leading cause of death among young children. The United Nations Resolution on global drowning prevention (2021) and World Health Assembly Resolution in 2023 have drawn attention to the issue. This scoping review synthesizes the current evidence on the effectiveness of child drowning prevention interventions since the 2008 World Report on Child Injury Prevention and implications for their implementation.

**Methods:**

Quantitative studies published between 2008 and 2023 focusing on interventions targeting unintentional injuries, including drowning, among children and adolescents under age 20 years were searched on Cochrane Database of Systematic Reviews, Epistemonikos, PubMed, and Embase. Relevant data on interventions were extracted using a pre-defined template on Microsoft Excel. This scoping review focuses on the interventions addressing drowning.

**Results:**

Overall, 12 studies fulfilled the inclusion criteria. Evidence generated between 2008 and 2023 support the effectiveness of introducing barriers around water bodies, immediate resuscitation and first-responder training, and use of personal floatation devices (PFDs). Basic swimming and water safety skills training for children ages 6 years and older, and enacting and enforcing regulations on pool fencing and PFD use were found to be promising based on new evidence published since 2008. This scoping review also found evidence on new interventions studied since 2008, such as close adult supervision, inspections of safety standards of pools, and the use of door barriers and playpens, all of which demand further research to ensure context-specific implementation in LMICs.

**Conclusion:**

While there is evidence to support both existing and new interventions, most of the available interventions are still classified as promising and emerging, underlining the need for further evaluation of those interventions in diverse settings (including low and middle- income) through effectiveness studies and implementation research. In addition, it is important to highlight the nexus between drowning prevention and the Sustainable Development Goals to advocate multisectoral and interdisciplinary collaboration, to influence the broader child health agenda.

## 1 Introduction

Drowning accounts for 9% of all injury-related deaths among people of all ages worldwide. More than 90% of these deaths occur in low- and middle-income countries (LMICs) (1, 2). Globally, drowning is one of the leading causes of death among children and adolescents under 20 years, leading to 95,947 deaths in 2019 alone (3, 4). Children ages 1–4 years are the most affected, followed by those ages 5–9 years (1). However, these estimates do not include drowning deaths due to flood disasters and water transport incidents, thus underestimating the true burden of drowning (1, 5). Moreover, data systems in LMICs do not regularly and reliably capture drowning data, leading to challenges in understanding and effectively addressing this burden (1).

Risk factors for drowning vary by context including age, access to water, poor socio-economic conditions, lack of swimming skills, lack of supervision of children and adolescents near water, living in geographically isolated and flood-prone locations, pre-existing medical conditions, such as epilepsy and autism spectrum disorder in children and adolescents, among others (1, 6–9).

There has been considerable progress in reducing the burden of drowning among all age groups globally, with estimates showing declining rates of drowning deaths across countries of varying income settings between 2000 and 2019, with significantly less improvement in low-income countries (reduction of 8% in low-income countries, 50% in lower-middle income countries, 29% in upper-middle-income countries and 46% in high-income countries (HICs)) (4). The decline in drowning deaths may be attributable to increased awareness of drowning as a major public health problem and increased commitment to drowning prevention from country governments, as well as the development and implementation of interventions targeted at preventing drowning (10).

Much of this attention and effort was stimulated by the World Report on Child Injury Prevention published by the World Health Organization (WHO) and United Nations International Children’s Emergency Fund (UNICEF) in 2008 (11). This report highlighted injury prevention strategies for five types of unintentional injuries including drowning across both HICs and LMICs. The report was based on evidence of the effectiveness of the prevention strategies, mainly from HICs. Since then, several advancements have been made in drowning prevention among children and adolescents, including the release of the “Global report on drowning: preventing a leading killer” in 2014 (2), the United Nations (UN) General Assembly adopting its first resolution on global drowning prevention in 2021 (12), and the adoption of a resolution by the World Health Assembly in 2023 seeking to accelerate action on drowning prevention globally (13).

In light of these advancements, a scoping review was deemed appropriate for synthesizing the literature on drowning prevention. Such reviews can also point to gaps in research and practice (14). Since 2008, only a few reviews have evaluated the effect of drowning prevention interventions on child mortality and morbidity (15, 16). The current scoping review synthesizes evidence on the effectiveness of drowning prevention strategies aimed at reducing drowning risk, mortality, and morbidity and improvement in intervention acceptability, water safety knowledge, and behavior among children and adolescents across both HICs and LMICs. This scoping review also categorizes interventions into different levels of effectiveness, and documents advancements that have taken place over a 15-year period since 2008 to improve their implementation. In addition, this scoping review highlights interventions that need further research and discusses some considerations for implementation.

## 2 Methods

Arksey and O’Malley’s framework was utilized to conduct this scoping review (17).

### 2.1 Research questions

This scoping review was guided by the following research questions.

- What is the current evidence of the effectiveness of drowning prevention strategies in reducing drowning risk, mortality, and morbidity and improving intervention acceptability, water safety knowledge, and behavior among children and adolescents since 2008?
- What are the levels of effectiveness (“effective or evidence-based,” “promising,” “emerging,” “ineffective,” and “harmful”) of new and existing interventions? How have the levels of effectiveness of interventions changed since 2008?

### 2.2 Searching for relevant studies

#### 2.2.1 Data sources and search strategy

This scoping review on child drowning prevention strategies is part of a broader review that focused on prevention strategies for unintentional injuries among children and adolescents under 20 years, such as road traffic injuries, drowning, burns, falls, choking/strangulation, and unintentional poisoning. Studies, namely, systematic and scoping reviews were searched for on the Cochrane Database of Systematic Reviews and Epistemonikos. The search strategy was planned in consultation with a librarian at the Johns Hopkins Bloomberg School of Public Health. The search teams used were “accidents,” “falls,” “drown,” “unintentional trauma,” “unintended trauma,” “road injury,” “car,” “automobile,” “autobus,” “bus,” “pedestrian,” “choking,” “strangulation,” “suffocation,” “burn,” “poisoning,” “falls” among others, in various combinations of MeSH (Medical Subject Headings) terms for children and adolescents under 20 years. The complete search strategy adopted can be found in Supplementary material 1. The use of relevant search terms for multiple types of injuries, along with drowning-specific terms, ensured inclusive search results on child injuries. The authors aim for this scoping review to be the first in the series of reviews focusing on child unintentional injuries.

#### 2.2.2 Citation management

EndNote X9 was used as a reference manager (18). Results from each database were downloaded to EndNote and duplicates were removed to get a master list for title and abstract review. Articles from the master EndNote library were imported into Covidence, an online software that facilitates the systematic screening of titles and abstracts, and full texts for the purpose of structured reviews (19). Covidence automatically removes duplicate articles and develops PRISMA (Preferred Reporting Items for Systematic Reviews and Meta-Analyses) diagrams depicting outcomes of the screening and extraction process.

### 2.3 Selecting studies

A stepwise approach to gathering evidence on child injury prevention interventions was undertaken with review of (a) systematic and scoping reviews published between 2008 and 2020, (b) primary studies published between 2020 and August 2023 and (c) hand-searched articles on drowning prevention.

#### 2.3.1 Eligibility criteria

The inclusion criteria included peer-reviewed literature namely, systematic, and scoping reviews focusing on the quantitative evaluation of effectiveness of interventions to reduce the burden of unintentional injuries (road traffic, drowning, burns, falls, choking/strangulation and unintentional poisoning) including broad domain of engineering, vehicle design, safety and equipment, legislation and standards, education and skills, management and adaptation. The search criteria were restricted to children and adolescents under 20 years and interventions tested and/or implemented between 2008 and 2023. Evidence from all geographic locations were captured.

We excluded studies that focused on population above 20 years, intentional injuries (self-harm, suicide, intentional poisoning, violence), and those that utilized qualitative methods. Grey literature and studies not in English were excluded.

While the eligibility criteria listed above applied to all unintentional injuries, this scoping review focuses on drowning only.

#### 2.3.2 Title and abstract screening (systematic and scoping reviews)

The complete list of articles was divided equally among four reviewers such that each article was reviewed by a single reviewer. The reviewers discussed articles that did not clearly follow the inclusion and exclusion criteria and a decision to include or exclude was made by group consensus.

#### 2.3.3 Full text screening and data extraction (systematic and scoping reviews)

The full text of each included article was downloaded to Covidence for simultaneous review and data extraction. Articles for which the full text was not readily available were requested from Johns Hopkins University library resources, and those that could not be procured were excluded.

A data extraction template with pre-defined variables including title, authors, publication year, journal name, year of the study, sample population, age range, study context and setting, injury types, interventions, outcome indicators, and findings, was set up in Microsoft Excel. Articles were divided among four reviewers who conducted data extraction independently. Any discrepancy in data extraction was discussed and resolved by group consensus.

#### 2.3.4 Title and abstract and full text screening (primary studies and hand-searched articles)

Following the review of scoping and systematic reviews, a top-up search was done on PubMed and Embase to include primary studies published between 2020 and August 2023. The full search strategy for primary studies can be found in Supplementary material 2.

The inclusion and exclusion criteria remained the same except for the inclusion of primary studies. Following the review of primary studies, some hand-searched articles that met the inclusion criteria were also included.

#### 2.3.5 Data extraction (primary studies and hand-searched articles)

Data from primary studies and hand-searched articles were extracted using the data extraction sheet described above.

### 2.4 Data analysis

All the extracted data were analyzed using deductive coding wherein the reviewers created tables synthesizing evidence on injury prevention interventions, including the context where they were delivered, study type, sample population, sample size, purpose of the study and relevant statistical evidence of effectiveness. The reviewers also assigned a level of effectiveness to each injury prevention intervention based on available statistical evidence using one of the five following categories. These categories were adapted from multiple sources. This included the 2008 World Report on Child Injury Prevention, 2007 Handbook of Injury and Violence Prevention, and consultations with experts in the field of injury prevention (11, 20). The five categories are described below:

- *“Effective” or evidence-based:* Programs or strategies that have peer-reviewed, documented empirical evidence of their effectiveness demonstrated through experimental trials, meta-analysis, or systematic review of experimental trials.
- *“Promising”*: Programs or strategies that have some scientific research or data showing positive outcomes related to prevention, but do not have enough evidence to support generalizable conclusions. These include empirical studies like quasi-experimental studies, studies conducted in only high-income settings, one-time studies irrespective of the context, and systematic review of all types of study designs.
- *“Emerging”* (labelled as “Insufficient evidence” in the 2008 World Report on Child Injury Prevention): Interventions that do not currently meet the definition of either effective or promising and further research is warranted. These included policy, expert opinion pieces, descriptive studies, case series, and case reports.
- *“Ineffective”*: Programs or strategies where documented empirical evidence indicated that they were not effective.
- *“Harmful”*: Interventions that may increase the risk of injury. These effectiveness levels were assigned based on the evidence found in this review.

Consultations with external stakeholders were not conducted for this scoping review.

## 3 Results

### 3.1 Systematic and scoping reviews

**Figure 1:**
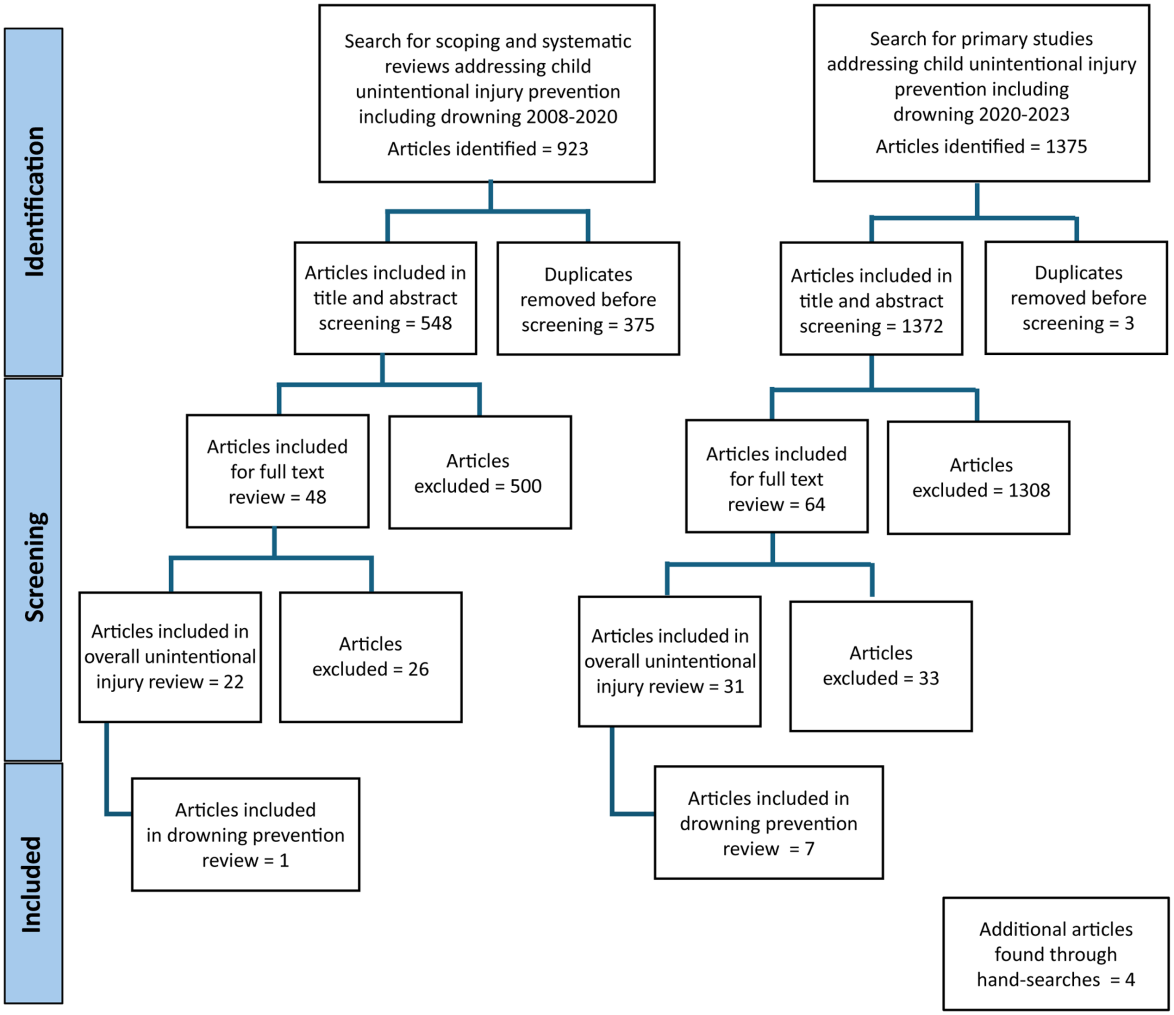
PRISMA flow diagram for the scoping review.

Based on the multi-injury search strategy, 923 systematic reviews and meta-analyses were identified. After duplicates (*N* = 375) were removed, a total of 548 articles were reviewed for title and abstracts. After title and abstract screening, 500 articles were found to be irrelevant. Of the 48 articles that were reviewed for full text, 26 articles were excluded (wrong outcomes = 9, interventions beyond the scope of the review = 6, hospital-based interventions = 5, adult population = 2, qualitative study design = 2, full text not available in English = 1, outcomes related to combined illnesses and injuries = 1). Twenty-two articles that met the inclusion criteria were included in the review on unintentional child injuries. Among the 22 studies included, 18 mentioned unintentional injuries other than drowning. Four studies mentioned child drowning in combination with other unintentional injuries, and of those only one presented statistical evidence of the effectiveness of drowning prevention interventions and was thus included in this scoping review (21) (Figure 1).

### 3.2 Primary studies

The top-up search of primary studies yielded 1,375 articles, of which three were duplicates and were removed. 1,372 articles were included for title and abstract screening, following which 1,308 articles were excluded. Sixty-four full texts were reviewed, of which 33 were excluded (wrong outcomes = 9, published before 2008 = 6, wrong study design = 6, full text not available = 5, no evaluation of effectiveness = 3, no intervention = 2, adult population = 1, wrong intervention = 1). Finally, 31 articles were included in the review on unintentional injuries, of which seven focused on drowning prevention interventions (15, 16, 22–26) (Figure 1).

### 3.3 Hand-searched articles

Five articles on drowning were found through hand searches, of which four evaluated the effectiveness of drowning prevention strategies and were thus included in the scoping review (27–30) (Figure 1).

### 3.4 Overall study characteristics

The scoping review included a total of 12 articles which provided statistical evidence of effectiveness for drowning prevention strategies. Of the 12, one was included from the review of systematic and scoping reviews, seven from the review of primary studies, and four from the hand-searched articles (two of which were technical reports). Among them, two focused on the global scenario of drowning prevention (21, 28), while three articles discussed interventions implemented in Bangladesh (23, 24, 29). Included articles also represent interventions evaluated in Australia (*n* = 3) (22, 27, 30), Malaysia (*n* = 1) (25), and the United States (*n* = 1) (26), while two systematic reviews discussed strategies adapted in multiple countries including, the United States and Australia (*n* = 1) (15) and the United States, Australia, Bangladesh, Greece, and Grenada (*n* = 1) (16) (Table 1).

**Table 1:** Characteristics of studies on drowning included in the scoping review.

Characteristic	Number (*N* = 12)
Study location	
Global	2
Australia	3
Bangladesh	3
Malaysia	1
United States	1
Multiple countries	2
Year of publication	
2008	1
2015–2017	2
2018–2023	9

**Figure 2:**
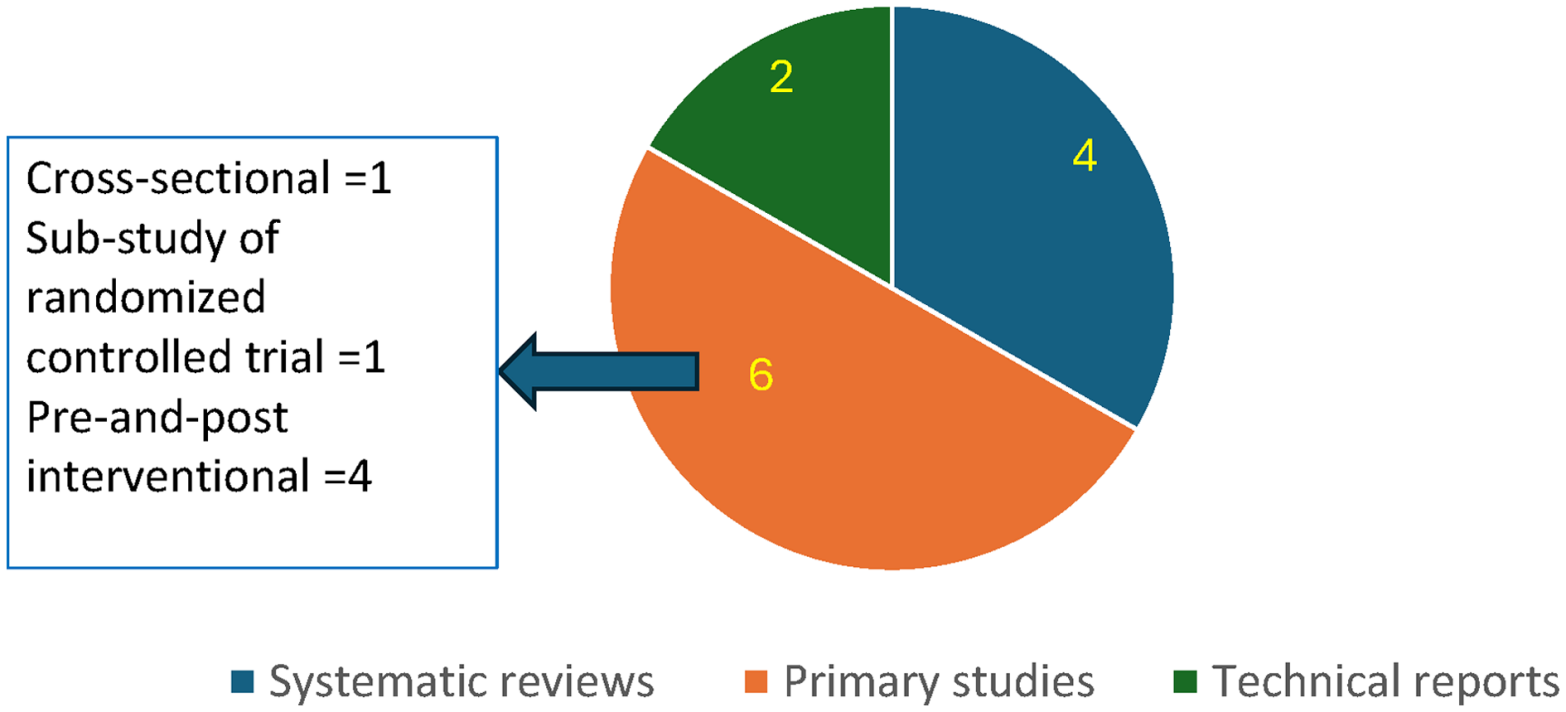
Types of studies included in the scoping review.

In terms of the type of studies, four systematic reviews (15, 16, 21, 22), six primary studies (23–27, 29), and two hand-searched technical reports were included (28, 30) (Table 1; Figure 2). While grey literature was excluded from this review, two technical reports were included, of which one provided an update on information regarding population at risk, water competency, and close supervision (28), and another (30) provided supporting statistical evidence for a systematic review (22) included in this review.

The timeframes for the systematic reviews varied (Table 1). The study population varied across the studies. These included children under 12 years (21, 27, 29), adolescents and young adults (16–25 years) (15, 16, 23, 28), parents with children under 10 years (24–26) and health care providers at children’s hospitals (26).

### 3.5 Drowning prevention interventions

Drowning prevention interventions included in this publication were classified as effective, promising, emerging, and harmful based on the evidence of effectiveness in reducing drowning risk, mortality, and morbidity among children and adolescents less than age 20 years, and improvement in intervention acceptability, water safety knowledge and behavior. No new evidence was found for interventions previously classified as harmful interventions in the 2008 World Report on Child Injury Prevention report (11). The changes in the effectiveness levels assigned to interventions since 2008 are shown in Table 2.

**Table 2:** Update on the level of effectiveness of drowning prevention interventions among children and adolescents under the age of 20 years since the 2008 World Report on Child Injury Prevention.

Drowning prevention interventions	Level of effectiveness	
	Year-2023	Year-2008
Removing (or covering) water hazards (31, 32)	Effective	Effective
Introducing barriers around water bodies (e.g., pool fencing) (15, 21, 28)	Effective*	Effective
Wearing personal floatation devices (11)	Effective	Effective
Ensuring immediate resuscitation (11, 23)	Effective*	Effective
Training on rescue and cardiopulmonary resuscitation (CPR) (23)	Promising	–
Ensuring the presence of lifeguards at swimming areas (11)	Promising	Promising
Community-based water safety education and awareness campaigns (15, 16, 24, 25, 27)	Promising	Ineffective
Basic swimming, water safety and safe rescue skills training for children 6 years or older** (15, 21, 22, 28)	Promising	Emerging
Enacting and enforcing legislation requiring the use of personal flotation devices (21)	Promising	Emerging
Enacting and enforcing legislation on pool fencing (28)	Promising	Emerging
Safety inspection of swimming pools (16, 28)	Promising	–
Close supervision (including in creches) (21, 29)	Promising	–
Use of door barriers and playpens (16, 29)	Emerging	–
Restricting access to areas unsafe for swimming (e.g., dams, wells, weirs, rainwater storage areas) (11)	Emerging	Emerging
Swimming lessons for children under age 6 years for improvement in motor skills** (15)	Emerging*	Emerging
Enacting and enforcing legislation on blood alcohol content for swimmers, boaters and those engaged in other water-based activities (11)	Emerging	Emerging
Promoting drowning prevention through doctors (26)	Emerging*	Emerging
Promoting solar pool covers as a drowning prevention strategy (11)	Harmful	Harmful
Using baby bath seats as a drowning prevention strategy (11)	Harmful	Harmful

*Level of effectiveness has not changed, but additional evidence has been found to support intervention. **Age for swimming lessons has changed from 5 to 6 years of age based on the WHO Guideline on the prevention of drowning through provision of day-care and basic swimming and water safety skills, 2021 (33).

#### 3.5.1 Effective interventions

A total of three studies discussed interventions that were found to be effective based on statistical evidence (15, 23, 28). Of these, one was a systematic review (15), one was a technical report (28), and the other was a primary study with a cross-sectional design (23). Although “effective” strategies were defined as those with empirical evidence of effectiveness from experimental studies or systematic reviews, interventions from the technical report (28) and cross-sectional study (23) were included under “effective” interventions as they provided additional supporting evidence on interventions already classified as “effective” in the 2008 World Report on Child Injury Prevention (namely, pool fencing and immediate resuscitation) (11).

##### 3.5.1.1 Removing (or covering) water hazards

Covering water hazards such as wells was found to be effective in the 2008 World Report on Child Injury Prevention (31, 32). The current scoping review did not find new statistical evidence of effectiveness published since 2008 (Table 2).

##### 3.5.1.2 Introducing barriers around water bodies (e.g., pool fencing)

Pool fencing was listed as an effective intervention in the 2008 World Report on Child Injury Prevention which is consistent with the findings of the current scoping review (Table 2). In the current scoping review, two studies discussed the effectiveness of pool fencing, a systemic review by Wallis et al. (15) and a technical report by Denny et al. (28). Both studies focused on high-income settings and children and adolescents under the age of 20 years (15, 28).

Wallis et al. discussed a specific study that explored the effects of domestic pool fencing using a case–control design to compare fatal and non-fatal drowning risk between four-sided fencing and unfenced pools or pools with three-sided fencing. The risk of drowning was statistically significantly higher in unfenced domestic pools compared to fenced domestic pools (15). Denny et al. referred to a study that compared four-sided domestic pool fencing with no fencing and found the number of immersion injuries to decrease by more than 50% among young children. Denny et al. also cited a Cochrane meta-analysis that found drowning risk in fenced pools to reduce significantly, compared to unfenced pools. The same analysis found four-sided fencing to be more protective compared to three-sided fencing (Odds Ratio, OR = 0.17 (95% CI 0.07 to 0.44)) (28) (Supplementary Table S1).

The primary study cited by Wallis et al., focused on fatal and non-fatal drowning events among children ages 0–13 years (15). Similarly, Denny et al. focused on children under age 14 years (28). Both studies included older children in the study population, while pool fencing has been shown to be effective specifically for children under age 3 years (15). In addition, pool fencing was only found to be effective when accompanied by a self-closing and self-locking gate (15, 28). Effectiveness of pool fencing is further affected by fence height and foot or handholds, view of the pool, and direction in which the self-closing gate should open (28). In addition, Wallis et al. highlighted that close supervision and basic swimming and water safety skills are necessary, in addition to pool fencing, for effective drowning prevention (15).

##### 3.5.1.3 Wearing personal flotation devices

Wearing PFDs was classified as an effective intervention in the 2008 World Report on Child Injury Prevention (11). The current scoping review did not find new statistical evidence on effectiveness published since 2008 (Table 2).

##### 3.5.1.4 Ensuring immediate resuscitation

Ensuring immediate resuscitation was classified as effective in the 2008 World Report on Child Injury Prevention which is in line with the findings of the current scoping review (11) (Table 2). Hossain et al., 2020 found cardiopulmonary resuscitation (CPR) provided immediately after rescue to lead to high survival rates (23) (Supplementary Table S1).

#### 3.5.2 Promising interventions

Overall, 10 studies presented evidence on “promising” interventions. Of these, four were systematic reviews (15, 16, 21, 22), two were quasi-experimental studies (25, 29), one was a cross-sectional study (23), one was a sub-study within a randomized controlled trial (24), one was a pre-and post-intervention study (27), and one was a technical report (28).

##### 3.5.2.1 Training on rescue and cardiopulmonary resuscitation

Training on rescue and CPR was not discussed in the 2008 World Report on Child Injury Prevention, however, based on new evidence this intervention was classified as “promising” in this scoping review. In the current scoping review, a primary study by Hossain et al. presented evidence on the effectiveness of community-volunteer led CPR of drowning victims (*N* = 82) in rural Bangladesh. The age of the drowning cases varied widely with 93% of the cases (*n* = 77) being under 18 years. Hossain et al. found that trained volunteers between 16 and 25 years, can provide first responder services to drowning casualties in the community. In the study, 31 drowning cases were treated with CPR, of which 22 survived and nine died. Higher post-CPR survival rate in this study was attributed to the fact that CPR was given immediately after casualties were rescued from the water (23) (Supplementary Table S1).

The study acknowledged contextual limitations of rolling out such interventions among rural populations in Bangladesh such as cultural hindrances in providing mouth-to-mouth breathing, and the assumption that drowning cases do not require medical treatment. In addition to these limitations, some volunteers did not report back to the study team about the cases they rescued and resuscitated, and the victims were not followed up to check for drowning-related morbidities following drowning events (23).

##### 3.5.2.2 Ensuring the presence of lifeguards at swimming areas

The presence of lifeguards at swimming areas was classified as a promising intervention in the 2008 World Report on Child Injury Prevention (11). The current scoping review did not find new statistical evidence of effectiveness of this intervention published since 2008 (Table 2).

##### 3.5.2.3 Community-based water safety education and awareness campaigns

Drowning prevention campaigns involving advertising hoardings were categorized as “ineffective” in the 2008 World Report on Child Injury Prevention (11). The current scoping review found educational and awareness campaigns to be promising (Table 2). A systematic review by Wallis et al. discussed media awareness campaigns in Washington, United States that promoted the importance of the use of PFDs and the supervision of children. This intervention resulted in increased PFD ownership and use (Supplementary Table S1). Prior to the campaign, 12 deaths occurred over a 3-year period among children 1–14 years, while eight deaths occurred in the 3-year period following the campaign. Without this intervention, the drowning death rate ratio in the program area would have been 0.58 (95% CI 0.21–1.58) following the trends in the rest of the state. Increased use of PFDs as a result of this awareness campaign was found to be associated with PFD ownership, parents’ age (<40 years), parents’ confidence in fitting a PFD, and their ability to recall the campaign. Although the target audience for this intervention was parents of children under age 14 years, it was delivered to the entire population (15).

In a systematic review by Leavy et al., one study reported increased PFD ownership among parents with children under the age of 14 years living in the United States, following a media campaign promoting PFD use and incentivization of PFD use with the provision of toys and stickers to children who used PFDs (Supplementary Table S1). However, the study population represented high-income families and thus these results may not be applicable to low-income families (16).

Hossain et al. explored the acceptability of phone-based text messages on drowning prevention among parents of children under the age of 5 years in a randomized trial in Bangladesh, as well as factors related to the acceptability of this form of knowledge dissemination. Overall, this method of spreading information on drowning was found to be acceptable. The acceptability of text messages was higher among males (76%), parents aged less than 30 years (80%), literate parents (76%), parents with a monthly income of more than 7,000 Bangladeshi taka (80%) and parents who had the ability to read SMS text messages (71%) (Supplementary Table S1). Analysis of costs associated with this phone-based intervention was missing from the study (24).

Farizan et al. conducted a quasi-experimental study in Malaysia to test the effectiveness of an educational intervention in improving parents’/guardians’ knowledge, and attitudes about drowning prevention and risk, and water safety practices. The study population included parents of primary school-aged children. Two intervention groups were included: provision of a health education booklet on drowning prevention and provision of the booklet plus a seminar about drowning prevention, while the control group received no education. Improvements in knowledge and attitudes were seen across all groups, with the greatest change seen among the booklet only group. The mean knowledge score for the booklet only group increased by 25% one month after the intervention, whereas for the booklet and seminar group scores increased by 22% (Supplementary Table S1). The post-intervention practice scores were lower than the pre-intervention ones, for all groups. These decreases were attributed to the limited duration of the study period (4 weeks) during which parents were unable to learn CPR skills accurately and the lack of prioritization of water safety skills training for children as a result of the cost associated with it (25).

Calverley et al. assessed water safety knowledge and competencies among children under ages 9 years and 12 years in Victoria, Australia, in addition to interviewing parents about their perception of their children’s lifesaving, water safety and survival swimming skills and knowledge. Water safety education included that related to signage recognition and interpretation, and safe behaviors in and around aquatic environments. Following the program, knowledge scores had a statistically significant mean increase from pre- to post-test (Supplementary Table S1). Parents appreciated the program since it helped develop an understanding of water safety in different aquatic environments and enabled the development of practical skills like rescue and first aid. Parents of children under age 9 years perceived their children’s lifesaving skills and knowledge to increase by 67% and those of children under age 12 years were perceived to increase by 74%. Water safety skills and knowledge of children under age 9 years and under age 12 years were perceived to increase by 23% and 24%, respectively. Survival skills and knowledge of children under age 9 years were perceived to improve by 43% while that of children under age 12 years were thought to improve by 6%. This study did not include any follow-up assessment of knowledge and skills, thereby the long-term impact of the program on knowledge and skills retention is unknown (27).

##### 3.5.2.4 Basic swimming, water safety and safe rescue skills training for children 6 years or older

Swimming lessons for children older than 5 years was classified as an emerging intervention in the 2008 World Report on Child Injury Prevention (11). Based on new evidence published since 2008, it was promoted to the “promising” level, and the age cut-off was changed to 6 years or older based on WHO’s recommendation in the “WHO Guideline on the prevention of drowning through provision of day-care and basic swimming and water safety skills (2021)” (33) (Table 2). A systematic review by Taylor et al. cited a study that evaluated a community-led basic swimming, water safety and rescue skills training program (including swimming and water safety instructions, training on “lifesaving” and first aid, installation of signage and distribution of water safety resources) delivered in remote areas across Australia (22, 30). The evaluation of this training program showed positive results for program coverage, parental satisfaction, and water safety skills (Supplementary Table S1). However, the training program targeted both children and adults. Implementation challenges associated with this intervention were difficulty in cultural appropriation in rural settings, lack of compliance with monitoring and evaluation steps among implementing organizations, and lack of ownership among project members who were not from the same community (30).

A systematic review by Vecino et al. found a combination of swimming lessons for children under age 14 years and supervision for children under age 5 years to be lifesaving (Supplementary Table S1). However, the authors acknowledged that the lives saved calculations made in their review assumed that the impact of interventions in HICs and LMICs would be the same (21).

Similarly, the technical report by Denny et al. cited a study that found swimming lessons for children ages 4–12 years reduced the risk of drowning, however, the estimates were imprecise and 95% CIs ranged from 3 to 99% (28). Wallis et al. saw a decline in drowning mortality rates among children ages 1–4 years who received formal swimming lessons, however, the association was not statistically significant (15) (Supplementary Table S1).

A primary study by Calverley et al. assessed water safety knowledge and competencies among children under ages 9 and 12 years in Victoria, Australia. The program titled “Bush Nippers” targeted drowning in in-land regions and aimed to improve water safety and lifesaving skills and knowledge through discussions and practical activities. Competencies included floating on back for 60 s, safe water entry and exit, out of water rescues, rescue elements (run-swim-run) and safe use of lifejackets and were assessed on a scale of 0–3 (0 = did not cover in program; 1 = did not attempt; 2 = participated, not competent; 3 = participated, competent). Overall, 71% of the children under age 9 years were competent in the practical skills, while 84% of the children under age 12 years were competent in these skills (Supplementary Table S1). This study did not include a baseline round of observations of competencies therefore no indication of program impact on aquatic competency could be determined (27).

##### 3.5.2.5 Enacting and enforcing legislation requiring the use of personal flotation devices

Legislation mandating PFD use was promoted to “promising” from its initial “emerging” level in the 2008 World Report on Child Injury Prevention (11) (Table 2). A systematic review by Vecino et al. found legislation mandating the use of PFDs in Victoria, Australia, reduced drowning mortality by 70% (RR = 0.30), however, the study did not include 95% confidence intervals for this measure of association (Supplementary Table S1). Additionally, this study did not focus on a specific age group, instead provided results for the “general population.” The study also acknowledged the limitation associated with scarce data on injury prevention interventions as a whole (21).

##### 3.5.2.6 Enacting and enforcing legislation on pool fencing

Pool fencing legislation was promoted from “emerging” to “promising” based on evidence found in this review (11) (Table 2). Denny et al. found pool fencing legislation to reduce the drowning death rates from 2.03 per 100,000 population 5 years prior to the implementation of legislation to 0.96 per 100,000 population in the 5 years post-implementation (Supplementary Table S1). Of note, methodological limitations of the study design used were not discussed in this article (28).

The results of pool fencing legislation may vary depending on the local understanding of “pool fencing,” which can range from four-sided pool fencing with self-closing and self-locking gates to alarming doors between homes and pool areas, and pool covers. This variation in how pool fencing is defined is an important consideration when implementing and evaluating the effectiveness of legislation mandating pool fencing. Additionally, pool fencing legislation is difficult to enforce, thereby affecting the effectiveness of these legislations (28).

##### 3.5.2.7 Safety inspection of swimming pools

Safety inspection of swimming pools was not discussed in the 2008 World Report on Child Injury Prevention, however, it was found to be promising based on new evidence (11) (Table 2). A systematic review by Leavy et al., included a study that found swimming pool inspections reduced fatal drowning events among children under age 20 years in the United States (Supplementary Table S1). However, this study did not present any data on non-fatal drowning and the decrease in drowning events may also be attributed to the increased presence, or CPR training, of lifeguards which happened simultaneously (16).

Another study cited by Leavy et al., explored the impact of government-led inspections of domestic swimming pools’ compliance with pool fencing legislation in homes with children under the age of 5 years in three councils (A, B, and C) of New South Wales, Australia (Supplementary Table S1). Increased compliance was observed in two councils (A and B), however, the study utilized convenience sampling methods. Also, two of the councils (B and C) selected had already had swimming pool inspections just before the study, so the compliance estimate for council B may be an overestimate (16).

##### 3.5.2.8 Close adult supervision

The current scoping review found new evidence of effectiveness of close supervision of young children by adults (11) (Table 2). A systematic review by Vecino et al. found close supervision at daycare centers or creches reduced drowning risk by 82% (95% CI 0.06–0.57) in Bangladesh and the authors estimated it could save more than 10,000 lives per year globally, specifically for children under age 5 years (21) (Supplementary Table S1).

A quasi-experimental pre- and post- intervention study in Bangladesh by Alonge et al. found creches significantly reduced drowning risk among young children in rural Bangladesh, where the interventions evaluated were creche only, playpen only, and both creche and playpen (Supplementary Table S1). Adult supervision was provided to children ages 9–36 months, who attended creches from 9 a.m. to 1 p.m., the time of the day during which they were at the highest risk of drowning. Although the study had a pre- and post- design, it enabled parents to choose which intervention they wanted for their children, and thus the study was not randomized and was subject to self-selection bias (29).

#### 3.5.3 Emerging interventions

A total of 4 studies provided evidence on “emerging” interventions. Of these, two were systematic reviews (15, 16), and two were primary studies (26, 29).

##### 3.5.3.1 Use of door barriers and playpens

Use of door barriers and playpens was not discussed in the 2008 World Report on Child Injury Prevention (11). The current scoping review found the provision of door barriers and playpens to be an “emerging” intervention as these interventions improve adult supervision (Table 2). However, since these interventions did not result in decreases in drowning risk, mortalities, or morbidities, the use of door barriers and playpens was classified as an “emerging” intervention.

A systematic review by Leavy et al. cited a study in Bangladesh where households with children ages 6–54 months were exposed to (1) educational messages only, (2) educational messages and a door barrier, or (3) educational messages and a playpen (an enclosure which limits children’s exposure to the external environment). Families that received a door barrier or playpen along with education left their children unsupervised less commonly, compared to those who received education alone (Supplementary Table S1). This study was not an effectiveness trial and utilized a convenience sample with no control group (16).

Another study in Bangladesh by Alonge et al. found playpens increased the risk of drowning (Supplementary Table S1). However, there were limitations cited in the study, such as different types of playpens being distributed in the two areas included so that the interventions were not equivalent, and the drowning risk between the two areas differing even without intervention, as one area was closer to rivers (29). Further research may be necessary to conclusively evaluate the effectiveness of playpens for drowning prevention.

##### 3.5.3.2 Restricting access to areas unsafe for swimming (e.g., dams, weirs, rainwater storage areas)

The 2008 World Report on Child Injury Prevention categorized restricting access to unsafe swimming areas as emerging since natural water bodies pose hazards associated with sudden changes in depth, strong currents, and low temperatures (11). The current scoping review did not find any new evidence on the effectiveness of this intervention (Table 2).

##### 3.5.3.3 Swimming lessons for children under age 6 years for improvement in motor skills

Swimming lessons for children under age 5 years was classified as an emerging intervention in the 2008 World Report on Child Injury Prevention (11). This is consistent with the findings of the current scoping review (Table 2).

A systematic review by Wallis et al. found swimming lessons for children between ages 1–4 years to reduce the rates of fatal drowning, however, the association was not statistically significant (adjusted OR = 0.12 (95% CI 0.01–0.97)) (15) (Supplementary Table S1). In addition, studies have shown that the learning period is much longer in younger children under age 2 years compared to older children, e.g., 6–10 years (11). Wallis et al. also included a study that found swimming lessons for children under age 5 years to improve motor skills for swimming, however, this study lacked a control group. This study also acknowledged that the positive results regarding swimming ability may have been due to familiarity with the water and not necessarily due to improvement in skill (15).

New evidence from the “WHO guideline on the prevention of drowning through provision of day-care and basic swimming and water safety skills (2021)” suggests a change in the age cut-off for swimming lessons for children aged 6 years or older (33). This highlights the need for more research around swimming lessons as a strategy for drowning prevention (11).

##### 3.5.3.4 Enacting and enforcing legislation on blood alcohol content for swimmers, boaters and those engaged in other water-based activities

The 2008 World Report on Child Injury Prevention categorized legislation mandating lower blood alcohol levels among those involved with water-based activities as emerging since there was a lack of statistical evidence of effectiveness of this intervention (11). The current scoping review did not find any new evidence on this intervention (Table 2).

##### 3.5.3.5 Promoting drowning prevention through doctors

Promoting drowning prevention through doctors was classified as an emerging intervention in the 2008 World Report on Child Injury Prevention, which is consistent with the findings of this review (11) (Table 2). In the current scoping review, a pre-and post-intervention study by McCallin et al. explored whether the frequency of counseling on drowning prevention in the United States increased when pediatricians were provided education and resources on the topic. The frequency of discussing water safety when families with children ages 0 to 10 years visited increased after the intervention. Pediatricians’ use of educational materials and knowledge about the burden and impact of drowning increased as well (Supplementary Table S1). However, the study relied on self-reported answers from pediatricians and thus may have been subject to recall and social desirability bias. In addition, some paper surveys were found to have incomplete data (26).

#### 3.5.4 Harmful interventions

The current scoping review did not yield new evidence on drowning prevention interventions classified as “harmful” in the 2008 World Report on Child Injury Prevention.

##### 3.5.4.1 Promoting solar pool covers as a drowning prevention strategy

No new evidence on solar pool covers was found in this scoping review (Table 2). The 2008 World Report on Child Injury Prevention described this intervention as harmful since children may get trapped under the pool covers and drown while they are left unsupervised. United States Consumer Product Safety Commission data from 1982 to 1988 showed 35 incidences of drowning associated with pool covers, among children under age 3 years (11).

##### 3.5.4.2 Using baby bath seats as a drowning prevention strategy

The current scoping review did not find any new evidence on the use of baby bath seats as a drowning prevention strategy (Table 2). The 2008 World Report on Child Injury Prevention classified this intervention as harmful since a case study in Australia reported 6 incidences of drowning among infants and children under age 2 years, who were placed in baby bath seats in adult bathtubs and left unsupervised. Similarly, another case study from the United States conducted over 13 years reported 32 drowning deaths among infants placed in bath seats (11).

## 4 Discussion

There has been a significant growth in the body of drowning literature since the release of the World Report on Child Injury Prevention in 2008. This includes the WHO’s 2014 release of the “Global report on drowning: preventing a leading killer” which described 10 actions for drowning prevention (2), the 2021 UN resolution that acknowledges the burden of drowning particularly among children in LMICs of Africa and Asia, and the need for drowning prevention to reduce child mortality to meet Sustainable Development Goal (SDG) 3 on health and well-being (12), WHO’s 2021 report emphasizing the importance of daycare centers and basic swimming and water safety skills training in LMICs (33) and the World Health Assembly (WHA) Resolution, “Accelerating action on global drowning prevention,” adopted in May 2023 (13). This scoping review is therefore timely since it synthesizes evidence on child and adolescent drowning prevention interventions discussed in the literature over a 15-year period, with a primary aim of providing an update on the effectiveness of drowning prevention interventions identified and implemented since 2008.

A majority of the interventions in this scoping review have been implemented in HICs. These interventions primarily focused on children and adolescents under age 20 years, with a specific focus on children under age 5 years. Pool fencing with self-latching and self-closing gates, immediate resuscitation, and first-responder training (including CPR) of young community volunteers were found to be effective. While covering wells and wearing PFDs are also classified as effective, no new evidence on their effectiveness, or new guidance on how to increase their uptake in LMICs where they are needed most, was found. Available evidence suggests that the presence of lifeguards at swimming areas, community-based educational programs, basic swimming, water safety, and rescue skills training, enacting and enforcing legislation for pool fencing and PFD use, inspections of safety standards of pools, and close adult supervision are “promising” strategies. However, there is still a lack of research on these interventions. Further efforts are needed to raise awareness of and build capacity for their adaptation, adoption and evaluation, particularly in LMICs.

Interventions that have been found to be successful in HICs may not be directly applicable or as effective in LMICs. Interventions such as pool fencing and pool fencing legislation, while effective, are more relevant to HICs. In LMICs, water sources like swamps and marshes appear on a seasonal basis or due to flooding (34). In addition, child drowning occurs during regular day-to-day activities like washing and bathing in ponds and canals near households in LMICs as opposed to recreational settings in HICs. Therefore, pool fencing and pool fencing legislation are not as relevant or effective in LMICs (35). In these settings, other interventions such as close adult supervision through daycare centers and basic swimming skills and water safety lessons have been shown to be promising and require greater attention to support their implementation (33). These have been recommended in the UN resolution on drowning prevention (12).

WHO recommends basic swimming skills and water safety training for children ages 6–10 years and categorizes this as having “moderate certainty in evidence” in terms of reducing drowning-related mortality, which is consistent with the findings of this scoping review. Supervision through daycare centers and basic swimming skills and water safety training target SDG 3.2 by aiming to reduce preventable deaths among children under age 5 years (33).

Interventions like swimming and resuscitation (e.g., CPR) training programs are associated with cultural limitations in LMICs and will need adaptation to local contexts and settings to support effective implementation (23). For instance, in rural settings, there is a heavy reliance on traditional rescue methods like spinning drowned children and putting pressure on the stomach, instead of utilizing safe, evidence-based resuscitation techniques like CPR (36). Similarly, in the case of swimming training programs, lack of participation among females has been observed due to cultural norms, menstruation among older female children, and involvement in domestic work. The same study also found female swimming instructors facing difficulty in managing classes during menstruation, since there were no protocols in place for the management of menstrual hygiene (37). These cultural contexts need to be taken into account when designing interventions for LMICs. For example, establishing sex-segregated training programs for female children that are led by female instructors and allowing participants to wear modest swimwear in line with their religious and cultural beliefs can address the lack of participation seen among women in LMICs (33, 36). Simultaneously, social behavior-change campaigns should be organized in local communities and Good Samaritan laws should be passed for the legal protection of first responders (2, 12).

This scoping review found regulations mandating use of PFDs to be effective in HICs (21). There is no empirical evidence on the effectiveness of the use of PFDs in LMICs, thus this area requires further research to determine settings and contexts in which mandated PDF use could be applied, e.g., ferry boat services or fishing industry (35).

Low-cost educational interventions that are interactive and focused, context-specific and appropriate for the target audience are most likely to be successful (15, 16). Based on the results of this scoping review, building community ownership through educational and awareness campaigns is recommended to ensure the success of implementing drowning prevention policies and programs in LMICs (12, 36).

While studies have explored the effectiveness of stand-alone interventions (such as close supervision in daycare centers, use of PFDs, and training programs addressing basic swimming, water safety, and rescue skills including CPR), implementing them individually will yield modest impact at best. Several studies suggest that effectively addressing the burden of drowning in LMICs will require implementing a combination of these strategies utilizing engineering, enforcement and/or educational measures, that will yield a synergistic effect at a larger scale (10, 15, 16). The daycare intervention in rural Bangladesh delivered in combination with community awareness raising efforts exemplifies combined prevention solutions and lays out a blueprint for a path towards successful implementation, scale-up, and sustainability (29). In addition to their benefits in preventing drowning, studies in Bangladesh have shown significant collateral benefits of daycares. Daycare centers in Bangladesh were found to be cost-effective, prevented other injuries, such as burns, falls, and poisoning, and resulted in enhanced psychosocial development in young children under age 2 years (38, 39). This has resonated with the Government of Bangladesh, which has taken up this strategy as part of its wider integrated early childhood development initiative and has committed approximately USD 25 million for a period of 3 years (38). These findings highlight the potential of situating drowning prevention within the broader context of child health and development and is a promising step in the right direction that other countries can adopt.

In addition to developing national, regional, and global prevention plans or strategies, drowning prevention needs to be integrated with broader public health agendas including that of climate change, and child and adolescent health (40). Furthermore, utilizing platforms such as the Sustainable Development Goals (SDGs) will enhance overall impact and sustainability of drowning prevention initiatives. This includes incorporating basic swimming and water safety training programs in the school curriculum that would ensure greater accessibility and equity to such programs while also advancing SDG 4.4 (2, 12). Similarly, it is necessary to set up surveillance systems for the collection of disaggregated fatal and non-fatal drowning data, including disaster-related drowning data, and this also addresses SDG 17.18 which focuses on collecting high-quality, reliable data (2, 12, 41). The cross-cutting nature of the problem demands multisectoral collaboration and knowledge sharing across sectors such as child health, education, environment, water and sanitation, and emergency response (40). In addition, collaboration among drowning prevention, disaster prevention and development experts is needed to manage the unique challenges associated with drowning events linked with climate change (40, 42).

Future research should focus on the effectiveness of the promising and emerging interventions discussed in this scoping review, in diverse settings, particularly in LMICs. For example, the effectiveness of embedding swimming, water safety, and rescue skills training in the school curriculum in improving retention of these skills should be tested. In addition, implementation research to understand the context in which interventions are delivered and the impact of combinations of interventions is necessary in LMICs to understand how to sustain and scale up these approaches to address the burden of drowning. Lastly, given the progressively devastating effects of climate change on health and environment, it is imperative to conduct exploratory research on the impact of climate change on drowning and roll out interventions that mitigate this impact (40).

Limitations and implementation challenges of individual studies included have been discussed in the “Results” section. This scoping review may have resulted in a reporting bias given it was limited to published English language, quantitative studies evaluating drowning prevention strategies. As such, evidence on behavioral aspects of effectiveness, including, community ownership, willingness to adopt prevention strategies, etc. as well as policy implications was limited. Primary studies published between 2008 and 2020 were not included unless they were part of a systematic or scoping review published during this period. However, most of the systematic reviews included in the current scoping review cover the latter part of this period, therefore this scoping review is likely to have captured relevant evaluations. In addition, hand searching for articles published between 2021 and 2023 yielded a few studies that matched the inclusion and exclusion criteria, namely that of studies focused on children and with quantitative results, therefore, this scoping review is not inclusive of all studies published during this period. However, recommendations from these studies were included in the discussion section.

## 5 Conclusion

This scoping review attempted to synthesize evidence of the effectiveness of child drowning prevention interventions that have been implemented globally between 2008 and 2023 for children and adolescents under age 20 years. Our scoping review showed that some positive progress has been made since 2008—some interventions that were classified as “emerging” in the 2008 report have moved to the “promising” category based on new evidence, and new promising and emerging interventions were also identified. However, the majority of this evidence is from HICs. While advancements in the field, including the UN resolution on drowning prevention, the World Health Assembly resolution to address the burden of drowning, and research around strategies for drowning prevention, represent a major leap forward for the field, much more needs to be done and data attained, to better understand what works, and how, in the diverse contexts represented by LMICs, where most child and adolescent drownings occur. Furthermore, as noted above, drowning prevention is a core component of the child health and development agenda, and strategies to prevent drowning have found health and development benefits beyond preventing drowning. This highlights the need for the drowning prevention community to develop linkages with other sectors beyond health including but not limited to water transportation, environment and climate, disaster risk reduction, water and sanitation, and migration, and engage in scholarly work around this issue to enhance its impact and implementation in countries and regions where children and adolescents are at greater risk of drowning.
