# Supplementary material for: Effectiveness of interventions to prevent drowning among children under age 20 years: a global scoping review
Source: Front Public Health. 2024 Dec 31;12:1467478. doi: 10.3389/fpubh.2024.1467478 (PMC11729736; doi:10.3389/fpubh.2024.1467478)
Supplement: Supplementary file 1 [file Data_Sheet_1.DOCX]

**Search for systematic and scoping reviews (2008-2020)**

Search – 2008 – 2020

Search engines – Cochrane Reviews, Epistemonikos

Types of articles - Reviews

Methodology – quantitative research methods

Population - Children (Under 20 years of age)

Other search terms

- Unintentional
- Injuries
- Interventions
- Effectiveness
- Road traffic injuries
- Drowning
- Burns
- Falls
- Poisonings
- Choking/strangulation
  - Engineering
  - Vehicle design
  - Safety Equipment
  - Legislation and standards
  - Education/ Skills
  - Management
  - Adaptation
  - Evaluation
  - Home
  - Community (School, playground)

| **Database** | **Hits** |
| --- | --- |
| Cochrane Reviews | 399 |
| Epistemonikos | 163 |
| Total without duplicates | 548 |

**Cochrane Library**

ID Search Hits: **399 Reviews**

#1 MeSH descriptor: [Accidents] this term only

#2 MeSH descriptor: [Accidental Falls] explode all trees

#3 MeSH descriptor: [Accidents, Home] explode all trees

#4 MeSH descriptor: [Accidents, Traffic] explode all trees

#5 MeSH descriptor: [Drowning] explode all trees

#6 MeSH descriptor: [Accidental Injuries] explode all trees

#7 ("Accident" OR "Accidents" OR "Accidental Falls" OR "Drown*" OR "Accidental Injur*" OR "unintentional injur*" OR "unintended injur*" OR "unintentional trauma" OR "unintended trauma" OR "traffic injur*" OR "road injur*" OR "car injur*" OR "automobile injur*" OR "autobus injur*" OR "bus injur*" OR "four-wheeler injur*" OR "two-wheeler injur*" OR "three-wheeler injur*" OR "pedestrian injur*" OR "bycycle injur*" OR "moped injur*" OR "motorcycle injur*" OR "cyclist injur*" OR "kombi injur*" OR "combi injur*" OR choking OR strangulation OR suffocation OR burn* OR scald* OR “hot water” OR steam OR poisoning OR fall*):ti,ab,kw

#8 #1 OR #2 OR #3 OR #4 OR #5 OR #6 OR #7

#9 MeSH descriptor: [Adolescent] explode all trees

#10 MeSH descriptor: [Child] explode all trees

#11 MeSH descriptor: [Infant] explode all trees

#12 ("Child" OR "Infant" OR "Adolescent" OR "Preschool" OR "children" OR "infants" OR "adolescents" OR "pediatric patient" OR "pediatric patients" OR "adolescence" OR "youth" OR "youths" OR "juvenile" OR "childhood" OR "teenager" OR "teenagers" OR "teen" OR "teens" OR "neonate" OR neonates OR "newborn" OR "newborns" OR "baby" OR "babies" OR "pediatric" OR "pediatrics" OR "paediatric" OR "paediatrics" OR "toddler" OR "toddlers" OR "under five" OR "under 5" OR "under 18" OR "under eighteen" OR "under 20" OR "under twenty"):ti,ab,kw

#13 #9 OR #10 OR #11 OR #12

#14 #8 AND #13

**Epistemonikos**

**Limited to January 1, 2008 to December 12, 2020**

<https://www.epistemonikos.org/en/>

**All: 260**

**Systematic Reviews, Structured Summaries, and Broad Syntheses: 163**

("Accident" OR "Accidents" OR "Accidental Falls" OR "Drown*" OR "Accidental Injur*" OR "unintentional injur*" OR "unintended injur*" OR "unintentional trauma" OR "unintended trauma" OR "traffic injur*" OR "road injur*" OR "car injur*" OR "automobile injur*" OR "autobus injur*" OR "bus injur*" OR "passenger injur*" OR "four-wheeler injur*" OR "two-wheeler injur*" OR "three-wheeler injur*" OR "pedestrian injur*" OR "bycycle injur*" OR "moped injur*" OR "motorcycle injur*" OR "cyclist injur*" OR "kombi injur*" OR "combi injur*" OR chok* OR strangulation OR suffocation OR burn* OR "hot liquid" OR steam* OR scald* OR poison* OR fall*)

AND

("Child" OR "Infant" OR "Adolescent" OR "Preschool" OR "children" OR "infants" OR "adolescents" OR "pediatric patient" OR "pediatric patients" OR "adolescence" OR "youth" OR "youths" OR "juvenile" OR "childhood" OR "teenager" OR "teenagers" OR "teen" OR "teens" OR "neonate" OR neonates OR "newborn" OR "newborns" OR "baby" OR "babies" OR "pediatric" OR "pediatrics" OR "paediatric" OR "paediatrics" OR "toddler" OR "toddlers" OR "under five" OR "under 5" OR "under 18" OR "under eighteen" OR "under 20" OR "under twenty" )

AND (prevent*)
