# Supplementary material for: Effectiveness of interventions to prevent drowning among children under age 20 years: a global scoping review
Source: Front Public Health. 2024 Dec 31;12:1467478. doi: 10.3389/fpubh.2024.1467478 (PMC11729736; doi:10.3389/fpubh.2024.1467478)
Supplement: Supplementary file 2 [file Data_Sheet_2.DOCX]

**Search for primary studies (2020-2023)**

| **Database** | **Hits** |
| --- | --- |
| PubMed | 1,387 |
| Embase | 1,688 |
| Total with dups removed—EndNote | 2,264 |
| Covidence total | 2,243 |

**PubMed**

**((("Accidents"[Mesh:NoExp] OR "Accidental Falls"[Mesh] OR "Accidents, Home"[Mesh] OR "Accidents, Traffic"[Mesh] OR "Drowning"[Mesh] OR "Accidental Injuries"[Mesh]** **OR** "Burns"[MeSH Terms] OR **"unintentional injur*"[tiab] OR "accidental injur*"[tiab] OR "unintended injur*"[tiab] OR "unintended trauma"[tiab] OR "unintentional trauma"[tiab] OR "traffic accident*"[tiab] OR "road accident*"[tiab] OR "car accident*"[tiab] OR "automobile accident*"[tiab] OR "autobus accident*"[tiab] OR "bus accident*"[tiab]** OR "four-wheeler injur*"[tiab] OR "two-wheeler injur*"[tiab] OR "three-wheeler injur*"[tiab]  **OR "pedestrian accident*"[tiab] OR "traffic injur*"[tiab] OR "road injur*"[tiab] OR "car injur*"[tiab] OR "automobile injur*"[tiab] OR "autobus injur*"[tiab] OR "bus injur*"[tiab] OR "pedestrian injur*"[tiab] OR** "bycycle injur*" [tiab] OR "moped injur*" [tiab] OR "motorcycle injur*" [tiab] OR "cyclist injur*" [tiab] OR "kombi injur*" [tiab] OR "combi injur*" [tiab] OR **drown*[tiab] OR “submersion injur*”[tiab] OR choking[tiab] OR chokes[tiab] OR choked[tiab] OR strangulation[tiab] OR suffocation[tiab] OR suffocates[tiab] OR suffocated[tiab] OR burn*[tiab]** OR scald*[tiab] OR “hot water”[tiab] OR steam[tiab] **OR poisoning[tiab] OR falls[tiab])**

**AND**

**("Child"[mh] OR "Infant"[mh] OR "Infant, Newborn"[mh] OR "Adolescent"[mh] OR "Child, Preschool"[mh] OR** **"child"[tiab] OR "infant"[all] OR "adolescent"[all] OR "children"[all] OR "infants"[all] OR "adolescents"[all] OR "pediatric patient"[all] OR "pediatric patients"[all] OR "adolescence"[all] OR "youth"[all] OR "youths"[all] OR "juvenile"[all] OR "childhood"[all] OR "teenager"[all] OR "teenagers"[all] OR "teen"[all] OR "teens"[all] OR "preschool child"[all] OR "neonate*"[all] OR "newborn*"[all] OR "baby"[all] OR "babies"[all] OR "pediatric"[tiab] OR "pediatrics"[tiab] OR "paediatric"[tiab] OR "paediatrics"[tiab] OR "toddler"[all] OR "toddlers"[all] OR "under five"[tiab] OR "under 5"[tiab] OR "under 18"[tiab] OR "under eighteen"[tiab])**

**AND ("prevention and control" [Subheading] OR "Accident Prevention"[Mesh] OR prevent*[tiab]))**

**NOT ("Qualitative Research"[Mesh] OR "qualitative research"[tiab] OR "qualitative stud*"[tiab] OR editorial[pt] OR letter[pt] OR case reports[pt] OR congress[pt] OR technical report[pt] OR news[pt] OR newspaper article[pt]))**

**AND (2019:2020[pdat])**

**Embase**

#7 #7 AND (2019:py OR 2020:py OR 2021:py)

#6 #3 AND #4 AND #5

#5 'prevention and control'/de OR 'control'/de OR 'medical countermeasure'/exp OR 'prevention'/de OR 'accident prevention'/exp OR prevent*:ti,ab,kw

#4 'juvenile'/de OR 'adolescent'/exp OR 'child'/exp OR 'child':ti,ab,kw OR 'infant':ti,ab,kw OR 'adolescent':ti,ab,kw OR 'children':ti,ab,kw OR 'infants':ti,ab,kw OR 'adolescents':ti,ab,kw OR 'pediatric patient':ti,ab,kw OR 'pediatric patients':ti,ab,kw OR 'adolescence':ti,ab,kw OR 'youth':ti,ab,kw OR 'youths':ti,ab,kw OR 'juvenile':ti,ab,kw OR 'childhood':ti,ab,kw OR 'teenager':ti,ab,kw OR 'teenagers':ti,ab,kw OR 'teen':ti,ab,kw OR 'teens':ti,ab,kw OR 'preschool child':ti,ab,kw OR 'neonate*':ti,ab,kw OR 'newborn*':ti,ab,kw OR 'baby':ti,ab,kw OR 'babies':ti,ab,kw OR 'pediatric':ti,ab,kw OR 'pediatrics':ti,ab,kw OR 'paediatric':ti,ab,kw OR 'paediatrics':ti,ab,kw OR 'toddler':ti,ab,kw OR 'toddlers':ti,ab,kw OR 'under five':ti,ab,kw OR 'under 5':ti,ab,kw OR 'under 18':ti,ab,kw OR 'under eighteen':ti,ab,kw OR 'under 20':ti,ab,kw OR 'under twenty':ti,ab,kw

#3 #1 OR #2

#2 'unintentional injur*':ti,ab,kw OR 'accidental injur*':ti,ab,kw OR 'unintended injur*':ti,ab,kw OR 'unintended trauma':ti,ab,kw OR 'unintentional trauma':ti,ab,kw OR 'traffic accident*':ti,ab,kw OR 'road accident*':ti,ab,kw OR 'car accident*':ti,ab,kw OR 'automobile accident*':ti,ab,kw OR 'autobus accident*':ti,ab,kw OR 'bus accident*':ti,ab,kw OR 'four-wheeler injur*':ti,ab,kw OR 'two-wheeler injur*':ti,ab,kw OR 'three-wheeler injur*':ti,ab,kw OR 'pedestrian accident*':ti,ab,kw OR 'traffic injur*':ti,ab,kw OR 'road injur*':ti,ab,kw OR 'car injur*':ti,ab,kw OR 'automobile injur*':ti,ab,kw OR 'autobus injur*':ti,ab,kw OR 'bus injur*':ti,ab,kw OR 'pedestrian injur*':ti,ab,kw OR 'bycycle injur*':ti,ab,kw OR 'moped injur*':ti,ab,kw OR 'motorcycle injur*':ti,ab,kw OR 'cyclist injur*':ti,ab,kw OR 'kombi injur*':ti,ab,kw OR 'combi injur*':ti,ab,kw OR drown*:ti,ab,kw OR 'submersion injur*':ti,ab,kw OR choking:ti,ab,kw OR chokes:ti,ab,kw OR choked:ti,ab,kw OR strangulation:ti,ab,kw OR suffocation:ti,ab,kw OR suffocates:ti,ab,kw OR suffocated:ti,ab,kw OR burn*:ti,ab,kw OR scald*:ti,ab,kw OR 'hot water':ti,ab,kw OR steam:ti,ab,kw OR poisoning:ti,ab,kw OR falls:ti,ab,kw

#1 'accident'/de OR 'home accident'/exp OR 'falling'/exp OR 'traffic accident'/exp OR 'drowning'/exp OR 'strangulation'/exp OR 'accidental injury'/exp OR 'burn'/exp OR 'suffocation'/exp OR 'airway obstruction'/exp

**Notes**: Emtree terms separate “suffocation” and asphyxia, only used “suffocation”.
