# Supplementary material for: Effectiveness of interventions to prevent drowning among children under age 20 years: a global scoping review
Source: Front Public Health. 2024 Dec 31;12:1467478. doi: 10.3389/fpubh.2024.1467478 (PMC11729736; doi:10.3389/fpubh.2024.1467478)
Supplement: Supplementary file 3 [file Table_1.docx]

**SUPPLEMENTARY FILE**

**Supplementary Table: Summary of evidence on interventions to prevent drowning among children and adolescents under 20 years since the 2008 World Report on Child Injury Prevention**

| **Author, study year, type of study and location** | **Sample population** | **Sample size** | **Purpose of study** | **Relevant results** |
| --- | --- | --- | --- | --- |
| Wallis et al., 2015 [15]  Systematic review  United States, Australia | Children and adolescents under 20 years | 7 studies | To analyze interventions designed to reduce fatal and non-fatal drowning events.  Statistical evidence of effectiveness of the following were presented:  -Educational campaigns aiming to improve life vest ownership and use  -Swimming and water safety lessons  -Pool fencing | Effect of education on PFD use:   - Children who owned a life vest were 1.60 times more likely to use a life vest compared to those without a PFD.   Effect of swimming and water safety lessons:   - Fatal drowning was less common among children 1-4 years who received formal swimming lessons (adjusted OR=0.12 (95% CI 0.01 to 0.97)) compared to those who did not. However, the association was not statistically significant. - Among children 5 to 19 years, swimming lessons reduced fatal drowning events by 64% (OR=0.36(95% CI 0.01 to 1.51)). However, the association was not statistically significant. - Swimming and water safety lessons (including deck behavior and water recovery) were effective in improving swimming ability for children 2-4 years (p<0.0001). However, it did not result in a statistically significant change in the rate of fatal drowning. This intervention is not feasible in many settings and cannot be used as a stand-alone intervention for drowning prevention.   Effect of pool fencing:   - The risk of drowning was 3.76 (95% CI 2.14 to 6.62) times higher in unfenced domestic pools than fenced domestic pools. |
| Denny et al., 2019 [28]  Technical report  HIC | Children and adolescents under 20 years | NA | To revise policy recommendations for effective drowning prevention interventions.  Statistical evidence of effectiveness of the following were presented:  -Pool fencing  -Pool fencing legislation  - Swimming and water safety and safe rescue skills training  -Safety inspections of swimming pools | Effect of pool fencing:   - Four-sided fencing was found to prevent >50% of swimming pool drownings in children under age 5 years. - A Cochrane meta-analysis found fencing to reduce drowning risk by 73% (OR= 0.27 (95% CI 0.16 to 0.47)), compared to unfenced pools.   Effect of pool fencing legislation:   - Pool fencing legislation reduced the rate of drowning deaths by half (from 2.03 per 100,000 population five years prior to the implementation of legislation to 0.96 per 100,000 population in the five years post-implementation) in the long term.   Effect of swimming, water safety and safe rescue skills training:   - Basic swimming, water safety, and safe rescue skills for children ages 4 to 12 years were shown to be effective in lowering the risk of drowning by 88%.   Effect of safety inspections of swimming pools:   - Government inspections of swimming pools resulted in compliance with pool fencing legislation to increase from 50% to 97%. |
| Hossain et al, 2020 [23]  Primary study (cross- sectional)  Bangladesh | Adolescents and youth volunteers 16-25 years | 2,305 | To describe the results of the volunteer-based first responder services for the management of drowned casualties in the rural communities of Bangladesh. | Effect of volunteer- based first responder training:  Those individuals who had a non-fatal drowning event had a survival rate of 71% if they received on average CPR for 16 minutes immediately after rescue by a first responder. |
| Leavy et al., 2016 [16]  Systematic Review  United States, Australia, Bangladesh, Greece, Grenada | Children 10 – 18 years | 15 studies | To analyze public health interventions designed to reduce fatal and non-fatal drowning and establish which child drowning prevention strategies are effective in preventing drowning in HIC and LMICs.  Statistical evidence of effectiveness for the following were presented:  -mass media campaigns  -comprehensive home safety parental education with or without a door barrier or playpen in 3 groups: (1) education alone, (2) education and a door barrier and (3) education and a playpen  -safety inspections of swimming pools | Effect of mass media campaigns:   - Reported PFD ownership increased by 75% following the campaign (n=230; 69% compared with n=361).   Effect of comprehensive home safety parental education alone, education and door barrier or education and playpen:   - Families who received only education left children unsupervised more commonly (6%) than families that received education and a door barrier (2%) or education and playpen (1%).   Effect of safety inspections of swimming pools:   - Swimming pool safety inspections for indicators such as, inadequate supervision, lack of barriers, poor water clarity, and failure to execute timely and competent rescue, resulted in a decrease in drowning events among children under 20 years from 0.21 per 100,000, in 1975-1976 to 0.02 per 100,000 in 1985-1986 in lifeguarded pools. - The number and rate of swimming pool submersions among children under 20 years also decreased from 1.06 per 100,000(n = 16) in 1974 to 0.48 per 100,000 (n = 7) in 1983). - Pool inspections resulted in increased compliance with regards to the criteria set for pool fence, gates, latch, access, and signage, from 49% to 55% in one council of New South Wales (NSW), while compliance increased to 97% in another council. |
| Hossain et al., 2020 [24]  Primary study (sub-study within a randomized controlled trial)  Bangladesh | Parents from 2 rural villages with at least one child under 5 years | 80 | To study the use of mobile phones and the factors related to the acceptability of text messages for parents for the prevention of child drowning in Bangladesh. | Effect of a mobile-phone based educational intervention:   - Of the respondents who had access to mobile phones, the majority (59/80, 74%) informed that they would like to receive phone-based short messaging service (SMS) text messages on drowning prevention. |
| Farizan et al., 2020 [25]  Primary study (randomized quasi-experimental pre-and post-intervention study)  Malaysia | Parents/guardians of primary school-aged children | 719 | To assess the effectiveness of a health education (booklet and seminar) intervention in improving parents/guardian’s knowledge on prevention of child drowning. | Effect of an educational intervention:   - The mean knowledge score for the booklet only group increased by 25% one month after the intervention, whereas for the booklet and seminar group scores increased by 22%. |
| Calverley et al., 2021 [27]  Primary study (pre- and post-intervention study)  Australia | Children under ages 9 and 12 years and their parents | 105 | To assess water safety knowledge and competencies among children under ages 9 years and 12 years, and parents’ perception of their children’s lifesaving, water safety and survival swimming skills and knowledge. | Effect of Bush Nippers program focused on water safety education and practical skills:   - Knowledge scores had statistically significant mean increase of approximately 5% from pre- to post-test (95% CI, 1.26-8.60), t(104) = 2.67, P = 0.009, d = 0.26. - 71% of the children under age 9 years were competent in the practical skills, while 84% of the children under age 12 years were competent in these skills. |
| Taylor et al., 2020 [22]  Systematic review  Australia | Children | 32 studies | To examine epidemiology, risk factors and prevention strategies of unintentional drowning by remoteness in Australia.  More specifically, the effectiveness of a community-led swimming, water safety and rescue skills training program in increasing coverage, parents’ satisfaction and swimming, water safety and rescue skills of children was explored. | Effect of swimming, water safety and rescue skills training program:   - 873 children were covered by the program, which exceeded the estimated number of 519.[30] - 99% of the parents expressed satisfaction with the program.[30] - ‘Lifesaving’ and water safety skills improved by a mean of 2.80 and 2.30 points respectively on a 10-point scale. However low performance was reported due to insuffient time for training.[30] |
| Vecino-Ortiz et al., 2018 [21]  Systematic Review  Poor countries (Categorized using the OPHI index); evidence on PFDs was extracted from a study in Australia; evidence on pool fencing was extracted from studies in Australia and New Zealand | General population | 47 studies | To review all the evidence on Effective interventions for the five main types of unintentional injury (road traffic injuries, drowning, falls, burns and poisoning).  To estimate the potential number of lives saved by effective injury interventions among the poorest billion.  Statistical evidence of effectiveness for the following were presented:  -PFD regulations  -Pool fencing legislations  -Swimming lessons and supervision | Effect of PFD regulations:   - Compulsory PFD wearing regulations reduced the risk of drowning deaths by 70% (Risk Ratio, RR=0.30) in Australia.   Effect of swimming lessons and supervision:   - Formal swimming lessons for children younger than 14 years and supervision for children younger than 5 years estimates > 25000 lives saved globally per year. - In Bangladesh, the use of creches for supervision of younger children under 5 years was also potentially effective with an estimated > 10,000 lives saved globally per year. |
| Alonge et al., 2020 [29]  Quasi-experimental pre- and post- intervention study  Bangladesh | Children under 5 years | 116,054 | To evaluate the large-scale effectiveness of creches and playpens in reducing the risk of drowning among children under 5 years | Effect of creches:   - Drowning risk was reduced in the post-intervention period among children under 5 years enrolled in a creche (cumulative incidence ratio = 0.12(95% CI 0.05 to 0.29)). - Children 2 and 3 years had 91% (cumulative incidence ratio = 0.09 (95% CI 0.02 to 0.36)) and 96% (cumulative incidence ratio = 0.04 (95% CI 0.002 to 0.60)) lower risk of drowning, respectively, compared to children under 1 year (cumulative incidence ratio = 7.00 (95% CI 0.85 to 57.83)).   Effect of playpen:   - The age-adjusted cumulative incidence ratio for children enrolled in the playpen intervention increased from 0 per 100,000 population per year in the pre-implementation period to 183.92 per 100,000 population per year in the post-implementation period. |
| McCallin et al., 2020 [26]  Primary study (pre-and post-intervention study)  United States | Caregivers for children 0-10 years and residents and attending providers at 2 children's hospitals | 103 caregivers and 83 residents | To engage pediatricians to increase the rate of drowning prevention counseling provided to families with children aged 0 to 10 years and increase knowledge of both providers and caregivers through education, resources, and materials geared toward drowning prevention. | Effect of provider education:   - Pediatricians' behavior in terms of discussing water safety during patient visits increased from 2% to 32%‚ after intervention. - Pediatricians' use of educational materials increased from 5% to 41%. - Pediatricians correctly answered that drowning is the second leading cause of death in children ages 1 to 14 years (64% pre-intervention to 95% post-intervention) and the awareness of brain damage from water-related injury increased (21% pre-intervention to 66% post-intervention). |
